# Low Levels of Human Antibodies to Gametocyte-Infected Erythrocytes Contrasts the PfEMP1-Dominant Response to Asexual Stages in *P. falciparum* Malaria

**DOI:** 10.3389/fimmu.2018.03126

**Published:** 2019-01-14

**Authors:** Jo-Anne Chan, Damien R. Drew, Linda Reiling, Ashley Lisboa-Pinto, Bismarck Dinko, Colin J. Sutherland, Arlene E. Dent, Kiprotich Chelimo, James W. Kazura, Michelle J. Boyle, James G. Beeson

**Affiliations:** ^1^Burnet Institute for Medical Research and Public Health, Melbourne, VIC, Australia; ^2^Department of Biomedical Sciences, School of Basic and Biomedical Sciences, University of Health and Allied Sciences, Ho, Ghana; ^3^Department of Immunology and InfectionLondon School of Hygiene and Tropical Medicine, London, United Kingdom; ^4^Center for Global Health and Diseases, Case Western Reserve University, Cleveland, OH, United States; ^5^Department of Biomedical Science and Technology Maseno University, Kisumu, Kenya; ^6^Department of Medicine, University of Melbourne Parkville, VIC, Australia; ^7^Department of Microbiology and Central Clinical School, Monash University Melbourne, VIC, Australia

**Keywords:** gametocytes, PfEMP1, antibodies, malaria, *P. falciparum*, immunity

## Abstract

Vaccines that target *Plasmodium falciparum* gametocytes have the potential to reduce malaria transmission and are thus attractive targets for malaria control. However, very little is known about human immune responses to gametocytes present in human hosts. We evaluated naturally-acquired antibodies to gametocyte-infected erythrocytes (gametocyte-IEs) of different developmental stages compared to other asexual parasite stages among naturally-exposed Kenyan residents. We found that acquired antibodies strongly recognized the surface of mature asexual-IEs, but there was limited reactivity to the surface of gametocyte-IEs of different stages. We used genetically-modified *P. falciparum* with suppressed expression of PfEMP1, the major surface antigen of asexual-stage IEs, to demonstrate that PfEMP1 is a dominant target of antibodies to asexual-IEs, in contrast to gametocyte-IEs. Antibody reactivity to gametocyte-IEs was similar to asexual-IEs lacking PfEMP1. Significant antibody reactivity to the surface of gametocytes was observed when outside of the host erythrocyte, including recognition of the major gametocyte antigen, Pfs230. This indicates that there is a deficiency of acquired antibodies to gametocyte-IEs despite the acquisition of antibodies to gametocyte antigens and asexual IEs. Our findings suggest that the acquisition of substantial immunity to the surface of gametocyte-IEs is limited, which may facilitate immune evasion to enable malaria transmission even in the face of substantial host immunity to malaria. Further studies are needed to understand the basis for the limited acquisition of antibodies to gametocytes and whether vaccine strategies can generate substantial immunity.

## Introduction

Strategies to develop highly effective vaccines against malaria remain a high priority. In particular, the development of vaccines that interrupt malaria transmission, known as transmission-blocking vaccines, is currently recognized as a key goal to sustain long-term malaria elimination (1). As such, there is a renewed interest in gametocytes, the sexual, transmissible stages of *Plasmodium falciparum*, which involves distinct parasite forms that establish infection in the mosquito vector. Currently, the advancement of transmission-blocking vaccines is hampered because very little is known about immune responses to sexual-stage antigens.

The sexual cycle of *P. falciparum* begins when immature asexual blood-stage parasites undergo commitment to produce gametocytes. The early gametocyte-IE stages (I–IV) are sequestered and develop within organs such as the spleen and bone marrow (2–4). Upon maturity to stage V, gametocyte-IEs are released into the peripheral circulation and taken up by feeding mosquitoes. During asexual development, *P. falciparum* remodels the host erythrocyte through the expression of knobs on the IE surface, which present the major surface antigen PfEMP1 (5). Specific interactions between PfEMP1 and host endothelial receptors enable the vascular sequestration of asexual parasites in various microvascular beds [reviewed in (6)]. However, knobs are absent from the surface of gametocyte-IEs (7), and PfEMP1 has not been detected on the surface of gametocyte-IEs, suggesting that gametocyte commitment is accompanied by the silencing of *var* genes (7). Other antigens have been identified on the surface of asexual IEs (including RIFIN, STEVOR, and SURFIN), with some evidence they are expressed by gametocyte-IEs (8).

Antibodies against circulating gametocytes have the potential to reduce malaria transmission efficiency by mediating parasite clearance within the human host or inhibiting further development of exflaggelated gametocytes within the mosquito midgut [reviewed in (9)]. However, knowledge of human antibodies against gametocyte-IEs is currently very limited [reviewed in (9)]. One study reported naturally-acquired antibodies recognized the surface of immature gametocyte-IEs (10). In contrast, other studies reported a lack of antibodies to immature stages, but some antibodies to mature stage V gametocyte-IEs (11–13). The target of these antibodies is unknown. In contrast, antibodies to gametocyte surface antigens, such as Pfs230 and Pfs48/45, are acquired relatively quickly and increase with cumulative exposure (14–18). As malaria transmission may still occur despite substantial acquired immunity to asexual parasites, it is likely that gametocyte-IEs do not share the surface antigens that elicit this immunity.

In order to address knowledge gaps in understanding human transmission-blocking immunity, we quantified antibodies to gametocyte-IEs compared to asexual IEs, and investigated the potential basis for differences in antibody reactivity to different developmental stages.

## Materials and Methods

A detailed description of methods is included in Supplementary Materials.

### Study Population and Ethics Statement

Plasma were collected at two study sites in Kenya (Kanyawegi and Chulaimbo) from individuals aged 0.5–79 years, as described (19, 20).

Ethics approval was obtained from Alfred Hospital Human Research and Ethics Committee, Australia, Institutional Review Board for Human Investigation at University Hospitals of Cleveland for Case Western Reserve University, USA and the Ethical Review Committee at the Kenya Medical Research Institute. Written informed consent was obtained from all study participants or their parents or legal guardians.

### *P. falciparum* Culture and Gametocyte Isolation

*P. falciparum* was maintained in continuous culture and synchronized as described (21). Isolates 3D7vpkd and 3D7-SBP1KO, with inhibited PfEMP1 surface expression, were generated as previously described (21, 22). Gametocytes were generated according to established protocols (11, 12), with the modification of using heparin (100 ng/mL) throughout gametocyte development to inhibit asexual replication (23).

### Measuring Antibodies to the IE Surface

Measuring IgG binding to the IE surface was performed by flow cytometry as previously described (21). IgG levels are expressed as the geometric mean fluorescence intensity (MFI; arbitrary units).

### Antibodies to Recombinant Proteins

We expressed a modified form of recombinant Pfs230D1H (24), a truncated form of Pfs230 containing the first 6-cys domain of Pfs230 (termed Pfs230D1M) expressed in the mammalian HEK293 cells. IgG binding to recombinant Pfs230D1M was measured using standard ELISA methods (25).

### Immunofluorescence Microscopy

Imaging of thin blood smears of stage V 3D7 gametocyte-IEs was performed as previously described (21) and processed using Photoshop CS6 (Adobe).

### Statistical Analyses

Non-parametric analytical methods were used to evaluate antibody results. Differences in antibody levels between trophozoite-IEs and gametocyte-IEs were assessed using a paired Wilcoxon signed rank test. Statistical analyses were performed using Prism version 7 (GraphPad Software Inc).

## Results

### Naturally-Acquired Human Antibodies to Gametocyte-IEs Are Markedly Lower Than to Asexual Trophozoite-IEs

**Figure 1:**
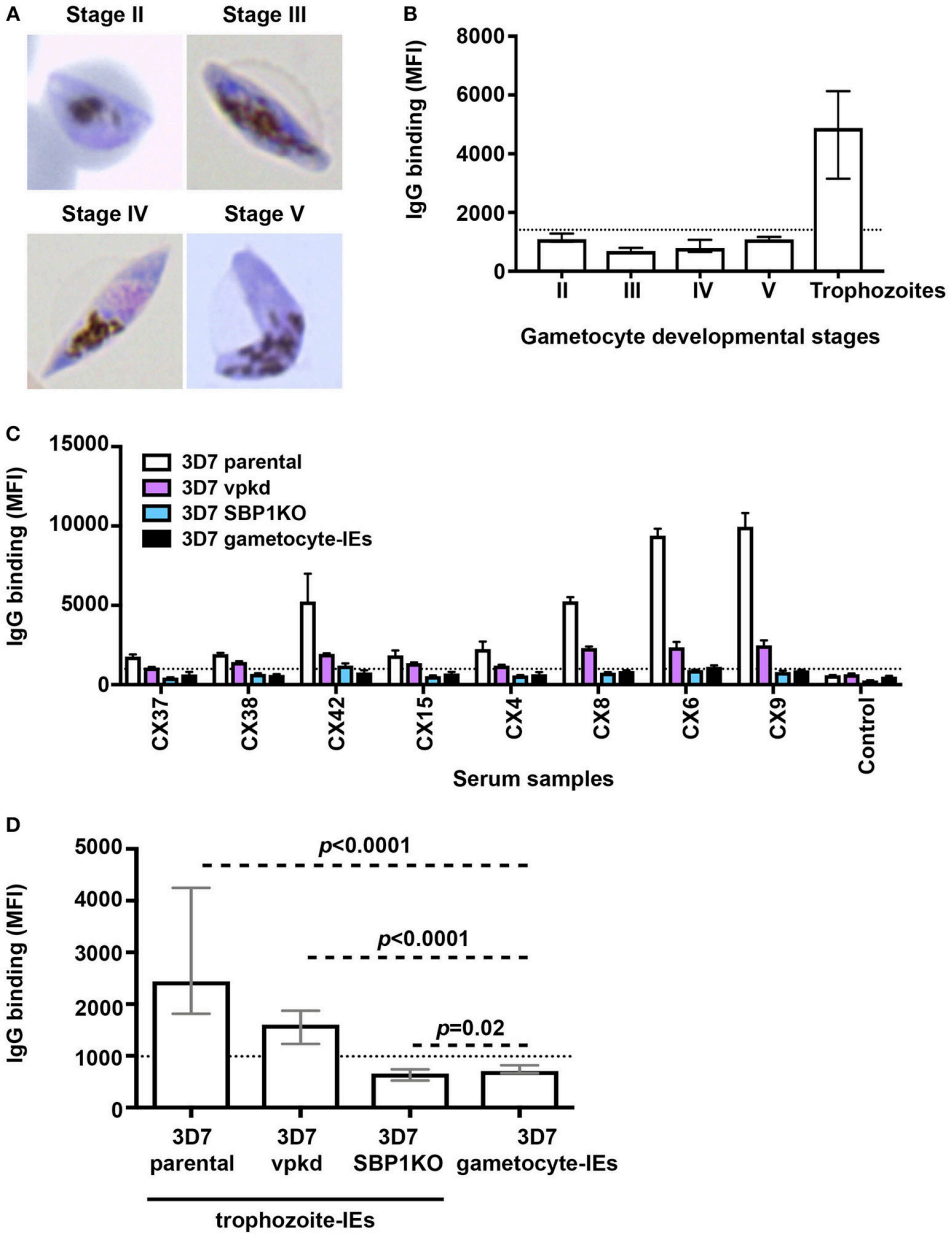
Low levels of naturally-acquired antibodies to the surface of gametocyte-IEs. **(A)** Giemsa-stained smears confirm the respective gametocyte-IE stages. **(B)** Total IgG binding to the surface of trophozoite-IEs and gametocyte-IEs was measured at stages II–V of gametocyte development. Samples were from malaria-exposed Kenyan individuals (children *n* = 11 and adults *n* = 10). The dotted line represents the antibody positivity threshold (MFI levels greater than mean + 3SD of non-exposed Melbourne controls). IgG binding levels are expressed as geometric mean fluorescence intensity (MFI) for all graphs; assays were performed thrice independently, with samples measured in duplicate (*n* = 21); bars represent mean and standard deviation. **(C)** A representative selection of plasma samples tested for antibodies to trophozoite-IEs and gametocyte-IEs. 3D7vpkd and 3D7-SBP1KO are transgenic parasite lines with inhibited PfEMP1 surface expression through the suppression of endogenous *var* genes (var promoter “knock-down”; vpkd) (21, 26) or genetic deletion of the PfEMP1 trafficking protein (skeleton-binding protein 1 “knock-out”; SBP1KO) (22, 27, 28); these were used at the asexual mature trophozoite stage. Samples were from malaria-exposed Kenyan individuals (CX; children *n* = 11 and adults *n* = 10) and non-exposed Melbourne residents (Control). IgG binding to gametocyte-IEs was substantially lower in all individuals compared to IgG binding to trophozoite-IEs. There was minimal background reactivity observed among sera from Melbourne residents; the dotted line represents the antibody positivity threshold. Assays were performed thrice independently; bars represent mean and range of samples tested in duplicate. **(D)** IgG binding to the surface of stage V 3D7 gametocyte-IEs was substantially lower compared to trophozoite-IEs of 3D7 parental and 3D7vpkd. The difference in IgG binding between gametocyte-IEs and trophozoite-IEs of 3D7 SBP1KO was minimal in our sample set. The dotted line represents the antibody positivity threshold (MFI levels greater than mean + 3SD of non-exposed Melbourne controls). Assays were performed thrice independently; bars represent median and interquartile ranges of samples tested in duplicate (*n* = 21; children *n* = 11 and adults *n* = 10); *p*-values were calculated using a paired Wilcoxon signed rank test.

We conducted a time-course assay based on the different developmental stages of gametocytes to assessed naturally-acquired antibodies. Thin blood smears were prepared and visualized by Giemsa staining to confirm the gametocyte stages (Figure 1A). We measured the level of human antibodies to the surface of stages II-V gametocyte-IEs by an established flow cytometry-based assay (21) in malaria-exposed individuals residing in Kenya (*n* = 21; children *n* = 11 and adults *n* = 10), compared to antibody binding to mature pigmented asexual trophozoite-IEs. There were noticeably low levels of antibody reactivity to the surface of gametocyte-IEs across all developmental stages (Figure 1B, Figure S1). Further, there was no significant difference in antibody levels observed between children and adults across different developmental gametocyte-IE stages (Figure S1F). In contrast, using the same selection of plasma samples (*n* = 21; children *n* = 11 and adults *n* = 10), high antibody reactivity was measured against surface antigens of trophozoite-IEs (Figure 1B, Figure S1). We confirmed our findings by measuring antibodies to stage V gametocyte-IEs in a second cohort of malaria-exposed Kenyan adults (*n* = 20; Figure S2). Similarly, all individuals had markedly lower antibody levels to stage V gametocyte-IEs compared to trophozoite-IEs (Figures S2A,B). Furthermore, increasing the plasma concentration from both human cohorts (1:2 dilution) used in assays did not substantially increase reactivity (Figures S2C,D). Our findings show that antibodies induced during natural malaria exposure have little reactivity to the surface of gametocyte-IEs across all stages.

### PfEMP1-Dominant Antibody Response in Asexual Trophozoite-IEs Contrasts the Low Response to Stage V Gametocyte-IEs

We hypothesized that the high reactivity to trophozoite-IEs, compared to gametocyte-IEs may be explained by antibodies targeting PfEMP1. Using genetically-modified *P. falciparum* with suppressed PfEMP1 expression, we found that PfEMP1 was a dominant target of naturally-acquired antibodies to the surface of asexual trophozoite-IEs (Figures 1C,D, Figure S3A) using the same selection of plasma samples from Kenyan children and adults (*n* = 21; children *n* = 11 and adults *n* = 10). This is consistent with our previous reports (21, 22). This was demonstrated by the greatly reduced reactivity of antibodies to 3D7vpkd and 3D7-SBP1KO IEs compared to 3D7 parental IEs. To better understand the magnitude of antibody responses to gametocyte-IEs, we compared antibodies to stage V gametocyte-IEs with asexual trophozoite-IEs that have reduced PfEMP1 expression (using 3D7vpkd and 3D7-SBP1KO). The level of antibodies to stage V gametocyte-IEs were substantially lower compared to trophozoite-IEs from 3D7 parental (71.1% lower; Figures 1C,D; *p* < 0.0001) and 3D7vpkd (55.9% lower; *p* < 0.0001). There was minimal difference in antibody levels to stage V gametocyte-IEs and 3D7-SBP1KO (*p* = 0.02). Prior studies suggested that there is still low levels of PfEMP1 expression on the surface of 3D7vpkd IEs (21, 22), which may explain the higher antibody reactivity to 3D7vpkd compared to 3D7-SBP1KO and gametocyte-IEs. These findings suggested that the lack of PfEMP1 on gametocyte-IEs is likely to be a major reason for the low reactivity of antibodies compared to trophozoite-IEs.

### Gametocytes Without the IE Membrane Are Recognized by Acquired Human Antibodies

**Figure 2:**
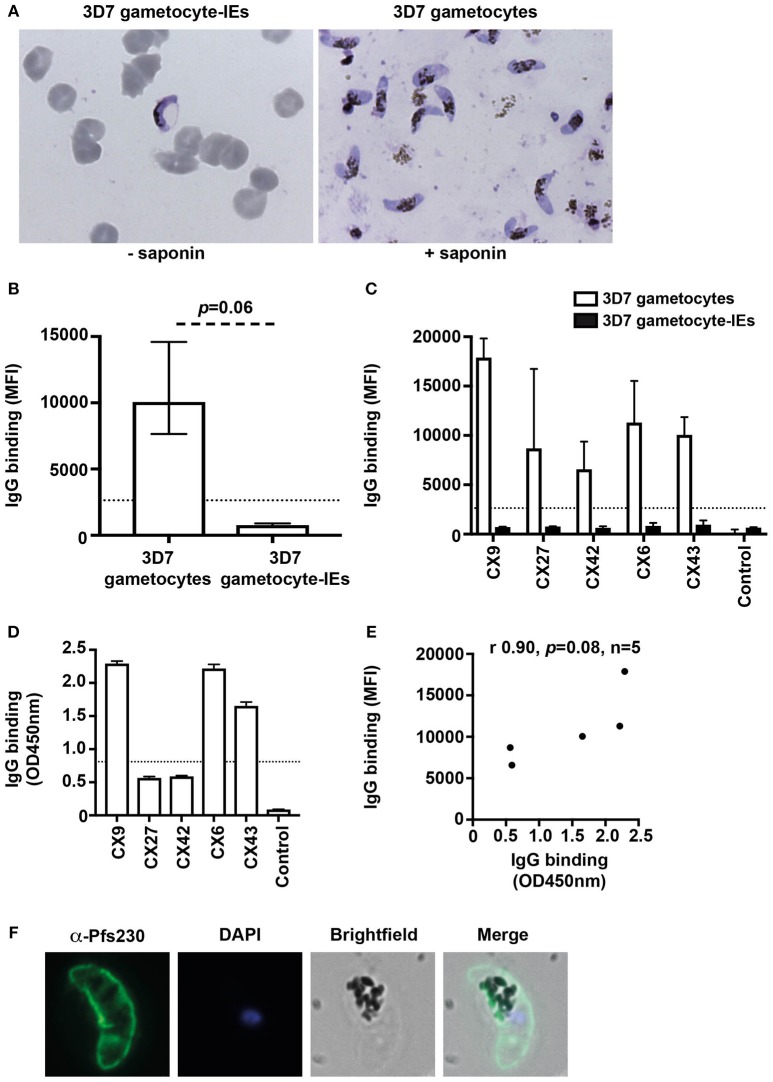
Antibodies recognize the surface of gametocytes in contrast to gametocyte-IEs. **(A)** Giemsa smears of gametocytes (without the erythrocyte membrane) and intact gametocyte-IEs that were used in antibody assays. **(B)** IgG binding to 3D7 gametocytes (without the erythrocyte membrane) was markedly higher compared to intact gametocyte-IEs. Assays were performed thrice independently; bars represent median and interquartile ranges of samples tested in duplicates (*n* = 5; children *n* = 3, adults *n* = 2); *p*-value was calculated using a paired Wilcoxon signed rank test. The dotted line represents the antibody positivity threshold (MFI levels greater than mean + 3SD of non-exposed Melbourne controls). **(C)** A representative selection of plasma samples tested for antibodies to 3D7 gametocytes and intact gametocyte-IEs. Samples were from malaria-exposed Kenyan individuals (CX; children *n* = 3, adults *n* = 2), and non-exposed Melbourne residents (Control). IgG binding to 3D7 gametocytes was substantially higher in all individuals compared to IgG binding to intact gametocyte-IEs. There was minimal background reactivity observed among sera from Melbourne residents; the dotted line represents the antibody positivity threshold. Assays were performed thrice independently; bars represent mean and range of samples tested in duplicate. **(D)** A representative selection of plasma samples were tested for total IgG binding to recombinant Pfs230D1M. The same selection of samples measured by flow cytometry (*n* = 21; children *n* = 11 and adults *n* = 10) was used. Antibody levels are expressed in optical density (OD) measured at 450 nm. Assays were performed twice; bars represent mean and range of samples tested in duplicate (*n* = 21); the dotted line represents the antibody positivity threshold. **(E)** There was a strong positive (non-significant) correlation between total IgG binding measured by flow cytometry (MFI) to 3D7 gametocytes and by ELISA (OD450 nm) to recombinant Pfs230D1M. Correlations were evaluated using Spearman's rho (r). **(F)** Immunofluorescence microscopy demonstrates the recognition of the native gametocyte surface by a Pfs230-specific antibody (green). Cells were fixed with 90% acetone and 10% methanol, and DAPI was used to stain nuclear DNA (blue). Representative images are shown.

We quantified the level of naturally-acquired antibodies to surface antigens expressed on the gametocyte plasma membrane. The IE membrane was removed by saponin treatment, and thin blood smears with Giemsa staining was used to confirm that gametocytes remained intact post-saponin treatment (Figure 2A). The level of antibodies to 3D7 stage V gametocytes and gametocyte-IEs was measured in a subset of Kenyan individuals (*n* = 5 from samples tested in Figure 1; children *n* = 3 and adults *n* = 2). High levels of antibodies were observed to 3D7 gametocytes, but not 3D7 gametocyte-IEs (Figures 2B,C, Figure S3B; reactivity 92.3% higher; *p* = 0.06).

To further assess antibodies to gametocytes, we measured antibodies to recombinant Pfs230, a major gametocyte surface antigen and vaccine candidate (24). We measured IgG reactivity in the same selection of Kenyan individuals used in assays of trophozoite-IEs and gametocyte-IEs (*n* = 21 from Figure 1; children *n* = 11 and adults *n* = 10). The majority of individuals (80.9%) were positive for antibodies to Pfs230D1M (Figure 2D; Figure S4) and there was a positive correlation between IgG binding to Pfs230D1M and to whole gametocytes (*r*_s_ = 0.9; Figure 2E). Further, immunofluorescence microscopy demonstrated that anti-Pfs230 rabbit antibody labeled native Pfs230 expressed on the surface of stage V gametocytes (Figure 2F). Together, these findings suggest that individuals do acquire antibodies to sexual stage parasites, but there is very limited acquisition of antibodies target antigens on the gametocyte-IE surface.

## Discussion

There was limited acquisition of antibodies to antigens on the surface of gametocyte-IEs, despite study subjects having high levels of antibodies to asexual trophozoite-IEs, as well as substantial antibody reactivity to the major gametocyte antigen, Pfs230. This finding was observed throughout different stages of gametocyte development. Our findings suggested that the lack of PfEMP1 on gametocyte-IEs may be a key explanation for the low antibody reactivity that contrasts the high reactivity to trophozoite-IEs. We demonstrated that the antibody response to asexual trophozoite-IEs is PfEMP1-dominant, with low levels of antibodies measured to genetically-modified trophozoite-IEs with suppressed PfEMP1 expression. Furthermore, antibody levels to gametocyte-IEs were comparable to trophozoite-IEs that largely lack PfEMP1. Interestingly, when the IE membrane was removed from stage V gametocytes, acquired antibodies had good reactivity to the gametocyte plasma membrane, indicating that the limited antibody reactivity to gametocyte-IEs was specific to IE surface antigens. Limited antibody acquisition to gametocyte-IEs may be an immune evasion strategy to enable malaria transmission to occur in the face of developing immunity (11, 15, 29, 30). The low or absent expression of PfEMP1 on gametocytes (8) may facilitate this immune evasion given that PfEMP1 is a dominant target of acquired immunity during blood-stage infection (6, 21, 22).

Naturally-acquired antibodies to trophozoite-IEs predominantly target PfEMP1 (21, 22), presented on the IE surface by knob structures (5). However, electron microscopy studies showed that gametocyte-IEs of all stages do not modify the IE surface with knobs, consistent with the absence of the essential knob component, KAHRP (7). Further, *var* gene transcription is downregulated at the onset of gametocyte differentiation, consistent with reports of the absence of detectable surface-exposed PfEMP1 in all stages of gametocyte-IEs (7). The lack of PfEMP1 expression on gametocyte-IEs likely explains the low antibody reactivity observed. As PfEMP1 is the main target of antibodies in asexual trophozoite-IEs, the downregulation of PfEMP1 in gametocyte-IEs may be an immune evasion mechanism to prevent clearance by antibodies. That gametocytes develop within erythrocytes, which lack MHC class-I, may further facilitate immune evasion. Furthermore, gametocyte development occurs predominantly in the bone marrow which may also be a factor influencing the acquisition of antibodies.

Interestingly, when the IE membrane was removed from the gametocytes, human antibodies recognized native antigens expressed on the gametocyte membrane itself, consistent with other reports on permeabilized gametocyte-IEs (13). Major gametocyte surface antigens include Pfs230 and Pfs48/45, which are targeted by human antibodies and associated with transmission-blocking activity through mosquito feeding assays [reviewed in (9)]. We found that the majority of samples tested in our study had high levels of IgG to recombinant Pfs230, suggesting that there was substantial acquisition of antibodies to gametocytes, but not gametocyte-IEs. Further, antibody levels measured to recombinant Pfs230 correlated with those against whole gametocytes.

Prior studies of gametocyte-IEs have reported some acquisition of antibodies mature stage V gametocyte-IEs (11, 12), although the prevalence of these antibodies was generally low. Differences in reactivity to stage V gametocyte-IEs between studies may relate to differences in sample populations and timing of sampling relative to infection episodes, including the presence of active gametocytemias. The focus of our study was to evaluate antibodies to gametocyte-IEs and understand the basis for the different reactivity between trophozoite-IEs and gametocyte-IEs. Future studies are needed to better understand antibodies to mature stage V gametocyte-IEs in different populations (age and geography) with different infection status and exposure history. It is possible that antibodies to gametocyte-IEs could be very short-lived, or antigens expressed could be transient in nature or weakly immunogenic, therefore requiring specific study designs to detect them. Our studies were limited to using the 3D7 *P. falciparum* isolate, and further work to evaluate antibodies to the surface of gametocyte-IEs of other isolates, especially recent clinical isolates, is warranted.

In conclusion, the limited acquisition of antibodies targeting gametocyte-IEs contrasts with the PfEMP1-dominant antibody response toward asexual trophozoite-IEs. Reactivity to gametocyte-IEs was comparable to the low antibody reactivity observed against trophozoite-IEs lacking PfEMP1. However, human antibodies were acquired against the surface of intact gametocytes and to Pfs230. The deficiency in acquired antibodies to gametocyte-IEs could be an important mechanism to avoid clearance by host immunity. Our findings provide new insights to address the major knowledge gaps in understanding immunity and malaria transmission, which will help inform the development of transmission-blocking vaccines.

## Ethics Statement

This study was carried out in accordance with the recommendations of Alfred Hospital Human Research and Ethics Committee, Australia, Institutional Review Board for Human Investigation at University Hospitals of Cleveland for Case Western Reserve University, USA and the Ethical Review Committee at the Kenya Medical Research Institute with written informed consent from all subjects. All subjects gave written informed consent in accordance with the Declaration of Helsinki.

## Author Contributions

J-AC and JB designed the study. J-AC conducted most of the experiments and analyzed the data together with JB. J-AC performed gametocyte culture with guidance from BD and CS. DD produced the recombinant Pfs230D1M protein. LR performed immunofluorescence microscopy. AL-P performed ELISAs. AD, KC, and JK were involved in cohort studies. J-AC, MB, and JB wrote the manuscript, which was critically reviewed by all authors. All authors approved the final manuscript.

### Conflict of Interest Statement

The authors declare that the research was conducted in the absence of any commercial or financial relationships that could be construed as a potential conflict of interest.
