# Supplementary material for: Low Levels of Human Antibodies to Gametocyte-Infected Erythrocytes Contrasts the PfEMP1-Dominant Response to Asexual Stages in P. falciparum Malaria
Source: Front Immunol. 2019 Jan 14;9:3126. doi: 10.3389/fimmu.2018.03126 (PMC6340286; doi:10.3389/fimmu.2018.03126)
Supplement: Supplementary file 1 [file Data_Sheet_1.docx]

**Chan et al**

**Low levels of human antibodies to gametocyte-infected erythrocytes contrasts the PfEMP1-dominant response to asexual stages in *P. falciparum* malaria**

**SUPPLEMENTARY MATERIALS**

**Supplementary Methods**

**Study population and ethics statement**

Plasma from malaria-exposed healthy, asymptomatic adults living in the Kanyawegi sub-district, Kisumu Country, Kenya (n=104, age range 18-79; AR) was collected in August 2004 (Supplemental Table S1) (19). Plasma from healthy, asymptomatic young children and adults living in the Chulaimbo sub-district, Kisumu County, Kenya (n=75, age range 0.3-5.9 years for children, age range 19.6-69.2 years for adults; CX) was collected in February-March 2007 (Supplementary Table S1) (20). Transmission intensity was relatively high at the time blood was collected (prevalence of asymptomic parasitemia in children ≤10 years was 70-80%) (30). Samples were screened for antibody reactivity towards the IE surface of mature 3D7 trophozoite-IEs and from this panel, samples selected for measuring antibodies to gametocyte-IEs were randomly chosen based on largest sample volume available for antibody measurement (children n=11 and adults n=10 from the Chulaimbo cohort, n=20 from the Kanyawegi cohort).

Ethics approval was obtained from the Alfred Hospital Human Research and Ethics Committee, Australia, the Institutional Review Board for Human Investigation at University Hospitals of Cleveland/Case Western Reserve University, USA and the Ethical Review Committee at the Kenya Medical Research Institute. Written informed consent was obtained from all study participants or their parents or legal guardians.

***P. falciparum* culture and gametocyte purification**

Asexual *P. falciparum* isolates were maintained in continuous culture and synchronized as previously described (21). Isolates 3D7vpkd (*var* promoter ‘knock-down’) and 3D7-SBP1KO (skeleton-binding protein 1 ‘knock-out’; SBP1KO) with inhibited PfEMP1 surface expression, were generated as previously described

(21,22). Gametocytes were generated from the 3D7 parasite line, according to established protocols using sorbitol synchronisation

(11,12), with the modification of using heparin (100ng/mL) throughout gametocyte development to remove residual asexual stages from the culture (23). Fully mature gametocytes were harvested 15 days post-induction for antibody assays. Stage V gametocyte-IEs were subjected to saponin lysis (0.15% saponin in RPMI for 5 min), which removes the IE membrane and parasitophorous vacuole membrane, leaving the parasite plasma membrane intact. For the time-course assay with all developmental gametocyte stages, parasites were harvested at days 3, 5, 7, 10 and 15 to reflect stages I-V of gametocyte growth and cryopreserved as described (21). Parasites were thawed as per established protocols prior to antibody assays (21).

**Expression of recombinant Pfs230D1M protein in HEK293F cells**

*Sequence selection and modification:*

Pfs230 is a member of the 6-cys family of proteins that include Pf12, Pf38 and Pf41. Pfs230 contains multiple 6-cys units, each containing up to 6 cysteine residues that form up to 3 disulphide bonded cysteine pairs. A short, truncated form of Pfs230 containing the first 6-cys domain of Pfs230 has previously been expressed as a recombinant protein in *P. pastoris* (Pfs230D1H)

(24). To assess naturally-acquired antibodies to Pfs230, we expressed a modified form of recombinant Pfs230D1H in the mammalian HEK293 cell expression system, which we termed Pfs230D1M. The Pfs230D1M protein sequence is based on the 3D7 allele of Pfs230 and contains amino acids Serine-542 to Glycine-726. When the sequence was assessed for potential glycosylation sites (http://www.cbs.dtu.dk/services/NetNGlyc/), an N-linked glycosylation motif was identified at Asparagine-585, and so this was substituted with a Serine. To mediate secretion of recombinant Pf230D1M protein into media and facilitate purification via nickel resin chromatography, a signal peptide for Tissue Plasminogen Activator (TPA), followed by a 6-histidine tag was fused to the N-terminus of the Pfs230D1M protein sequence.

*Generation of protein expression vectors:*

The Pfs230D1M protein sequence was used to reverse translate a DNA sequence that was codon-optimized for mammalian expression and then synthesized (GeneArt). The synthetic Pfs230D1M gene was supplied in a Puc vector, and then cloned into pcDNA 3.1+ using *Xho*1 and *Bam*H1 restriction sites. The final plasmid was quantified and used to transfect HEK293F cells.

*HEK293F cell culture and transfection:*

HEK293 Freestyle^TM^ cells (Thermo Fisher Scientific) were cultured following the manufacturer protocols. In brief, cells were cultured in Erlenmeyer shaker flasks (125ml, Corning) with FreeStyle™ 293 Expression Medium (Thermo Fisher Scientific) at 37°C, 8% CO_2_ at 135rpm on an orbital shaker. Cells were counted using the trypan blue (0.4%; Thermo Fisher Scientific) cell exclusion method using *Countess^TM^* Cell Counting Chamber Slides (Thermo Fisher Scientific) and the Countess^TM^ automated cell counter (Thermo Fisher Scientific). HEK293F cells were transfected for protein expression following the manufacturer’s protocol (Thermo Fisher Scientific) with minor alterations. On the day of transfection, cells were centrifuged (700*g* at 4°C, 10 min) and resuspended in HEK293F expression media with 1:100 antibiotic/anti-mycotic solution (Thermo Fisher Scientific) at a final density of 1x10^6^ cells/ml. For a 30ml transfection, 90µl of Polyethylenimine (PEI) transfection reagent (25 kDA linear; Polysciences; stock 1mg/ml) was added to 0.6 ml of OptiPro^TM^ Serum Free Medium (Thermo Fisher Scientific) and incubated for 5 minutes. Then this solution is added to the DNA solution (30µg of purified plasmids and 0.6ml of OptiProTM Serum Free Medium (Thermo Fisher Scientific)). After incubation for 10 min at room temperature, this final solution is added to the cells, and then returned to the orbital shaker and incubator. The next day, Lupin (1:40 of 20% w/v, Biotech Solabia) and Pluronic acid F-68 (1:100 of 10 % w/v) (Thermo Fisher Scientific) was added. The expressed protein was harvested 6 days post-transfection by centrifuging cells (700*g,* 10 min) to collect supernatant that was then filtered (0.2µm membrane) and stored at 4°C until purification. Protein expression was evaluated by SDS-PAGE gels and Western blot analyses.

*Pfs230D1M protein purification and dialysis:*

The harvested media containing the expressed Pfs230D1M protein was passed over nickel resin columns (Life Technologies), washed with 20mM imidazole/PBS (Sigma-Aldrich), and bound protein eluted in several 1ml fractions in 500mM imidazole /PBS. Eluted fractions were tested for the presence of protein by spectroscopy and SDS-PAGE. Fractions containing protein were pooled, filter sterilized, dialyzed into sterile PBS, and adjusted to a concentration of 1mg/ml via centrifugation using 10,000MW cut-off filters. Protein size and purity was confirmed by SDS-PAGE.

**Measuring antibodies to the IE surface by flow cytometry**

Measuring total IgG binding to the IE surface of pigmented trophozoites was performed with an established flow cytometry-based assay as previously described (21). Briefly, IEs were sequentially incubated with test plasma (1/10 dilution), polyclonal rabbit anti-human IgG (1/100) and AlexaFluor 488-conjugated donkey anti-rabbit IgG (1/500) with ethidium bromide to distinguish between infected and uninfected erythrocytes (1/1000), with washing between each step (Supplemental Fig S5). All incubations were conducted for 30min at room temperature and samples were measured in duplicates. Serum samples from malaria-naïve Australian donors were used as negative controls, while samples from malaria-exposed individuals were used as positive controls in all assays. Data was acquired by flow cytometry (FACS Verse, BD Biosciences) and analysed using FlowJo software (example of gating strategies are presented in Supplemental Fig S5). Levels of IgG specific to IE surface antigens in each plasma sample were expressed as the geometric mean fluorescence intensity (MFI; arbitrary units) for IEs, after subtracting that for uninfected erythrocytes. Plasma from malaria-exposed adults was used as positive controls. IEs used in flow cytometry assays were either fresh from culture or cryopreserved, and thawed prior to use.

**Measuring antibodies to recombinant Pfs230D1M by ELISA**

Measuring total IgG binding to recombinant Pfs230D1M was performed using standard ELISA methods as previously described (25). Briefly, Pfs230D1M was coated onto Maxisorp microtiter plates (Nunc) at 0.5µg/ml. Plasma samples were tested at 1/100 for total IgG, followed by a secondary polyclonal goat anti-human IgG HRP-conjugated antibody used at 1/1000 (Life Technologies). TMB liquid substrate (Life Technologies) was used to measure enzymatic reactivity and the reaction was stopped with 1M sulphuric acid. PBS was used as a negative control and plates were washed thrice using PBS with 0.05% Tween in between antibody incubation steps. The level of antibody binding was measured as optical density at 450nm.

**Immunofluorescence Microscopy**

Thin blood smears of stage V 3D7 gametocyte-IEs were prepared as previously described (21). Briefly, slides were blocked in 1% BSA in PBS for 30 min and incubated with antibodies from rabbits immunised with Pfs230D1M (R1873; 1/100), followed by an AlexaFluor 488-conjugated antibody (1/500). Images were collected using a Plan-Apochromat (100×/1.40) oil immersion phase-contrast lens (Carl Zeiss) on an AxioVert 200M microscope (Carl Zeiss) equipped with an AxioCam Mrm camera (Carl Zeiss). Images were processed using Photoshop CS6 (Adobe).

**Statistical analyses**

Non-parametric analytical methods were used to evaluate antibody results from human cohort studies as the data generated are not normally distributed. Differences in antibody levels between trophozoite-IEs and gametocyte-IEs were assessed using a paired Wilcoxon signed rank test. Statistical analyses were performed using Prism version 7 (GraphPad Software Inc). Correlations between antibody levels measured by flow cytometry and ELISA were evaluated using Spearman’s rho (r_s_). Antibody positivity threshold is defined as IgG binding levels (measured as MFI by flow cytometry or at OD 450nm by ELISA) that are greater than the mean+3SD of non-exposed Melbourne controls.

**Supplementary Figures and Tables**

**Figure S1 Selection of individual plasma samples measured to all stages of gametocyte-IEs**

**A**. Total IgG binding to the surface of trophozoite-IEs and gametocyte-IEs were measured at stages II to V of gametocyte development. Samples were from malaria-exposed Kenyan individuals (CX; children n=11 and adults n=10). The dotted line represents the antibody positivity threshold (MFI levels greater than mean+3SD of non-exposed Melbourne controls). IgG binding levels are expressed as geometric mean fluorescence intensity (MFI) for all graphs; assays were performed thrice independently, with samples measured in duplicate (n=21); bars represent mean and standard deviation.

**B-E**. A representative selection of plasma samples tested for antibodies to surface of 3D7 asexual stage trophozoite-IEs compared to all developmental stages of gametocyte-IEs. Samples were from malaria-exposed Kenyan individuals (CX; children n=11 and adults n=10) and from non-exposed Melbourne residents (Control). IgG binding to gametocyte-IEs was substantially reduced in all individuals compared to IgG binding to trophozoite-IEs. There was minimal background reactivity observed among sera from Melbourne residents. IgG binding levels are expressed as geometric mean fluorescence intensity (MFI) for all graphs; assays were performed twice independently; bars represent mean and range of samples tested in duplicate (n=21).

**F**. Total IgG binding to the surface of gametocyte-IEs were measured in the same selection of samples of Kenyan adults (n=10) and children (n=11). No difference in antibody binding was observed between adults and children in all stages of gametocyte development. Error bars represent median and interquartile ranges; *p* values were calculated using an unpaired Mann Whitney test.

**Figure S2 Low levels of naturally-acquired antibodies to the surface of gametocyte-IEs in plasma from Kenyan individuals**

**A**. A representative selection of plasma samples tested for antibodies to surface of 3D7 asexual stage trophozoite-IEs or sexual-stage gametocyte-IEs. Samples were from malaria-exposed Kenyan adults (AR; n=20) and from non-exposed Melbourne residents (Control). The overall level of IgG binding to gametocyte-IEs was substantially reduced in all individuals compared to IgG binding to trophozoite-IEs. There was minimal background reactivity observed among sera from non-exposed Melbourne residents. IgG binding levels are expressed as geometric mean fluorescence intensity (MFI) for all graphs; assays were performed twice independently; bars represent mean and range of samples tested in duplicate (n=20).

**B**. IgG binding to the surface of 3D7 gametocyte-IEs was substantially reduced compared to trophozoite-IEs. The dotted line represents the median of non-exposed Melbourne controls. Assays were performed twice independently; error bars represent median and interquartile ranges of samples tested in duplicate (n=20); *p* value was calculated using a paired Wilcoxon signed rank test.

**C**, **D**. IgG binding to the surface of trophozoite-IEs and gametocyte-IEs was measured at a higher concentration of 1:2, compared to previous assays that were tested at 1:10. Increasing plasma concentrations did not impact the level of antibody recognition to the surface of gametocyte-IEs.

**C**. IgG binding to the surface of 3D7 gametocyte-IEs was substantially lower compared to trophozoite-IEs. Assay was performed once; bars represent median and interquartile ranges of plasma samples tested in singles (CX, n=6 with children n=3 and adults n=3; AR, adults n=6).

**D**. A representative selection of plasma samples tested for antibodies to 3D7 trophozoite-IEs and gametocyte-IEs. Samples were from malaria-exposed Kenyan children and adults from two study sites in Kenya (CX, n=6 with children n=3 and adults n=3; AR, adults n=6) and from non-exposed Melbourne residents (Control). IgG binding to gametocyte-IEs was substantially reduced in all individuals compared to IgG binding to trophozoite-IEs. There was minimal background reactivity observed among sera from Melbourne residents. IgG binding levels are expressed as geometric mean fluorescence intensity (MFI) for all graphs; assay was performed once; bars represent the MFI values.

**Figure S3 Low levels of naturally-acquired antibodies to the surface of gametocyte-IEs compared to trophozoite-IEs without PfEMP1**

**A**. IgG binding to the surface of stage V 3D7 gametocyte-IEs was substantially lower compared to trophozoite-IEs of 3D7 parental and 3D7vpkd. The difference in IgG binding between gametocyte-IEs and trophozoite-IEs of 3D7 SBP1KO was minimal. Samples were from malaria-exposed Kenyan individuals (children n=11 and adults n=10). The dotted line represents the antibody positivity threshold (MFI levels greater than mean+3SD of non-exposed Melbourne controls). *p* values were calculated using a paired Wilcoxon signed rank test.

**B**. IgG binding to 3D7 gametocytes (without the erythrocyte membrane) was markedly higher compared to intact gametocyte-IEs. Samples were from malaria-exposed Kenyan individuals (children n=3, adults n=2); *p* value was calculated using a paired Wilcoxon signed rank test.

**Figure S4 Kenyan individuals with low antibody levels to gametocyte-IEs had high antibody levels to Pfs230D1M**

The same selection of plasma samples used to measure antibody binding to the surface of gametocyte-IEs (at stages II-V of development) were used to measure antibody binding to recombinant Pfs230D1M. Samples were from malaria-exposed Kenyan individuals (CX; children n=11 and adults n=10) and non-exposed Melbourne residents (Control). Antibody levels are expressed in optical density (OD) measured at 450nm. Assays were performed twice; bars represent mean and range of samples measured in duplicate (n=21).

**Figure S5 Gating strategy for flow cytometry analyses of IEs**

Representative flow cytometry plots from assays that measure IgG binding to the surface of trophozoite-IEs (left) and gametocyte-IEs (right) using serum from a Kenyan adult (Chulaimbo cohort). The y-axis represents the level of Alexa488 fluorescence, which is a measure of IgG binding and the x-axis represents the level of ethidium bromide (EtBr) fluorescence, which is a measure of parasitemia. In each plot, uninfected erythrocytes are gated on the left (EtBr negative) and infected erythrocytes are gated on the right (EtBr positive). Within the population of infected erythrocytes, there was a higher level of IgG binding to trophozoite-IEs compared to gametocyte-IEs (Alexa488 positive cells).

**Table S1 Summary of the number of plasma samples used for antibody testing**

|  | Kanyawegi (AR) | Chulaimbo (CX) |
| --- | --- | --- |
| Children | - | 11 |
| Adults | 20 | 10 |
